# Effect of COVID-19 pandemic on mental health hospital admissions: comparative population-based study

**DOI:** 10.1192/bjo.2021.975

**Published:** 2021-08-03

**Authors:** Fabian Bonello, Daniela Zammit, Anton Grech, Victoria Camilleri, Rachel Cremona

**Affiliations:** Department of Psychiatry, Mount Carmel Hospital, Malta; Department of Psychiatry, Mount Carmel Hospital, Malta; Clinical Chairman of Mental Health Services, Mount Carmel Hospital, Malta; and Senior Lecturer, Department of Psychiatry, Medical School, University of Malta; Department of Psychiatry, Mount Carmel Hospital, Malta; Department of Psychiatry, Mount Carmel Hospital, Malta

**Keywords:** COVID-19, pandemic, suicidality, hospital admission, population-study

## Abstract

**Background:**

The coronavirus disease 2019 (COVID-19) global pandemic caused mental health services to be downscaled to abide by the public health restrictions issued.

**Aims:**

The aim of this study was to investigate whether the pandemic and resultant restrictions had an impact on Malta's admissions to hospital for mental health issues by assessing the number and nature of psychiatric admissions to our only national mental health hospital.

**Method:**

Data collection was carried out retrospectively for the 13-week period between 7 March 2020 and 4 June 2020, compared with the equivalent in 2019. Demographic data was obtained and descriptive statistical analysis through the use of the χ²-test, *z*-test and logistic regression model were used to compare both data-sets, using a *P*-value of 0.05.

**Results:**

An overall reduction in admissions to hospital was noted in 2020 when compared with 2019, recorded to be lowest in March 2020 with a steady acceleration of admissions up until May 2020 (χ^2^(3) = 22.573, *P* < 0.001). This coincided with a decelerated rate of positive COVID-19 cases locally. In 2020, there were significantly higher female admissions (χ^2^(1) = 10.197, *P* < 0.001), increased presentations of self-harm/suicidal ideation (*P* < 0.001) and higher involuntary admissions using the Mental Health Act (χ^2^(1) = 4.904, *P* = 0.027). The logistic regression model identified total length of stay in hospital, primary mental health diagnosis, gender and month of admission as variables significantly associated with an admission.

**Conclusions:**

Our first population-wide study confirms that the COVID-19 pandemic and subsequent public health restrictions had an impact on the population's hospital admissions for mental health issues.

The first case of SARS-CoV-2 infection, more commonly known as coronavirus disease 2019 (COVID-19), was reported in Wuhan, a city in the Hubei province in China, in December 2019. Less than a year later, it has reached pandemic status, causing a staggering total of 158.7 million active infections worldwide with 3.3 million deaths reported at the time of writing.^1^

In Malta, the first positive case was registered on the 7 March 2020,^2^ with public health authorities immediately issuing hand hygiene and physical distancing directives. On 20 March 2020, a decision was made to downscale mental health services such that they could cope with the increasing limitations being posed by this crisis while providing adequate ongoing care to the population. This included the cessation of all non-urgent out-patient psychiatric services as well as the closure of the Short Stay Psychiatric Unit offered through the national general medical hospital (Mater Dei Hospital), with an increased investment in care provision through community mental health clinics, primarily via telepsychiatry. A new service (the admissions and referrals team) was also set up with the aim of mitigating psychiatric admissions that could be managed in the community, through improved support of primary healthcare professionals and closer communication with community mental health teams. Emergency/liaison psychiatry and admissions to the national mental health facility (Mount Carmel Hospital) remained available at all times.

A week later, on 27 March 2020, the local public health authorities, among other restrictions, decided to implement a partial lockdown confining those who were deemed vulnerable (i.e. those with chronic medical conditions and/or ≥65 years of age) at home. Patients with severe mental illness were not included in the vulnerable group. These restrictions remained in force until 5 June 2020, where following a steady decrease in the infection rate, a decision was made to start scaling back restrictions.^2^ This time period represents the first wave of COVID cases in Malta.

## Implications on mental health

Considering the magnitude, its widespread effect and its relatively unknown nature, it was expected that this pandemic would have an impact on the population's mental health. It has caused public panic and mental health stress,^3^ but its effect is more far reaching than that. The World Health Organization predicted an increase in loneliness, insomnia, anxiety, depression, harmful drug and alcohol use, self-harm and suicidal behaviour.^4^ Increased home confinement may result in an increase in cases of domestic violence as women and children have no respite from their abusers in isolation.^5,6^ Population-based surveys in the USA and China have in fact confirmed a number of these predictions.^7–9^ It is in consideration of the above that the aim of this study was to investigate whether the COVID-19 pandemic had an impact on the nation's mental health hospital admissions, using the number and nature of psychiatric admissions as a measure.

## Method

The study evaluated all admissions to the only national mental health facility in Malta, which caters for a population of around 500 000. This study excludes the population of the sister island of Gozo, which amounts to around 25 000 people, and is catered for by a small psychiatric unit in their regional hospital who receive any psychiatric presentations warranting admission. Data collection was carried out retrospectively for the 13-week period between 7 March 2020 and 4 June 2020. This represents the period of time between the first case of COVID-19 recorded locally, up until the day prior to the discontinuation of a number of the major public health restrictions (5 June); a period that is considered as the first wave of the pandemic in Malta. Data for the equivalent time period in 2019 was collected in an attempt to remove, or at least mitigate, the effect of seasonality.

Information was gathered using the electronic admissions register that provided patient demographics, including age, gender and length of stay. Physical in-patient files and discharge letters were used to obtain clinical details including primary mental health diagnosis, mode of admission and use of the Mental Health Act. All identifiable patient data was anonymised prior to data analysis to ensure protection of all sensitive and personal details. In both years, some admissions were noted to have incomplete data. For this reason, inferential statistics were completed only where the data were available.

Data analysis was carried out using the IBM Statistical Package for the Social Sciences (SPSS) version 25. Descriptive statistics were presented graphically using line graphs and clustered bar graphs. The difference between two proportions *z*-test and the χ²-test was used to compare the prevalence of a number of primary mental health diagnoses (expressed as percentages) between March and June in 2019 and 2020. For both tests, a 0.05 level of significance was adopted. Moreover, to analyse all variables collectively, a logistic regression model was fitted to identify which of the six categorical variables, (i.e. month of admission, age groups, gender, the primary mental health diagnosis, length of stay and use of Mental Health Act) had a significant impact on the number of admissions between the two time phases (2019 and 2020).

Regarding ethical approval, an approval form was obtained from the Data Protection Office of Mount Carmel Hospital for the collection, analysis and publication of retrospectively obtained and anonymised data for this non-interventional study.

## Results

### Number of admissions

**Fig. 1:**
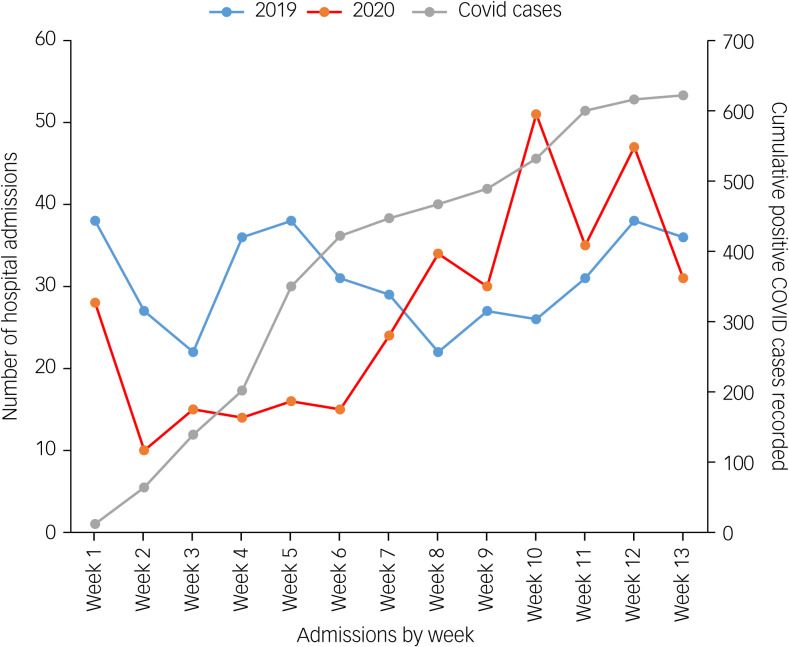
Total number of admissions occurring every week in both years, in comparison with positive COVID cases recorded in Malta in 2020.

A comparison of the total number of hospital admissions occurring between March and May 2019 (*n* = 401), with the same time period in 2020 (*n* = 350) found there were 49 admissions less in 2020 during the COVID-19 pandemic. Despite the reduced numbers, there was a sharp increase in the rate of admissions from March to May 2020, with the highest percentage of admissions occurring in May 2020 (weeks 9–12) surpassing that of the previous year, as shown in Fig. 1.

This figure also compares the number of admissions by week between March and June 2019 and 2020, compared with the cumulative number of confirmed COVID positive cases being recorded in Malta for the same time period in 2020. As the rate of COVID positive cases started to plateau around week 6, at the same time the number of admissions in 2020 started to increase (see Supplementary Table 1 in the Supplementary material available at https://doi.org/10.1192/bjo.2021.975). The χ²-test was used to compare the total number of hospital admissions between March and June in both years. The test reveals that there was a significant difference in the monthly admissions between March and June in 2019 and 2020 (χ^2^(3) = 22.573, *P* < 0.001).

### Demographics

The demographic factors of interest for the admissions in question were primarily age and gender. The most common age groups admitted in both years remained those between 18 and 40 years of age, i.e. younger adults, constituting around half of the total admissions. However, in 2020, one can notice a higher proportion of admissions of older adults, namely over 60 years of age (11.5% in 2019 *v*. 16.9% in 2020). The *P*-value of the χ²-test (0.063) exceeds the 0.05 level of significance by a small margin, indicating that the percentage change is considerable but not significant.

In both years, the number of male admissions exceeded female admissions. Nevertheless, the percentage of female admissions increased from 28.9% to 40% during the COVID period in 2020, whereas male admissions decreased from 71.1% to 60%. This change in admissions by gender (11.1%) is significant at the 0.05 level of significance (χ^2^(1) = 10.197, *P* < 0.001).

### Primary mental health diagnoses

**Fig. 2:**
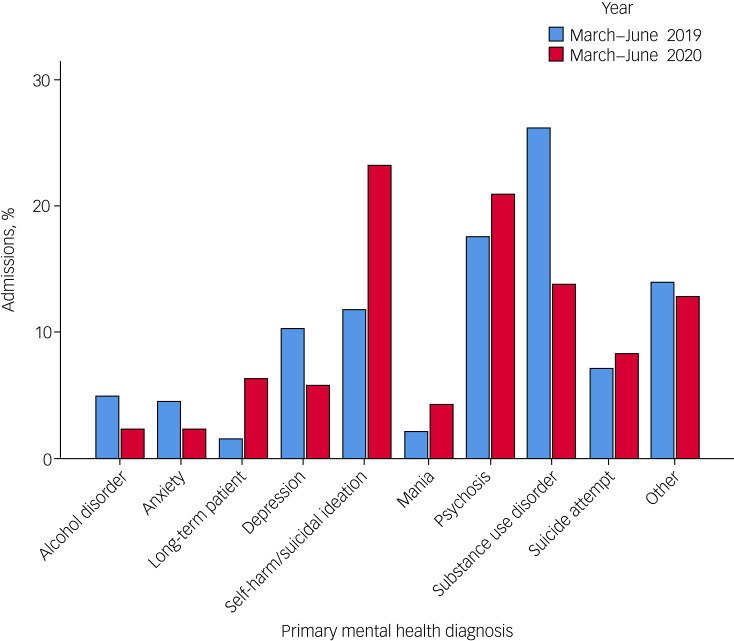
Percentage admissions in both years, by type of primary mental health diagnosis.

Table 1 and Fig. 2 display the percentage of admissions according to their primary mental health diagnosis in both time periods. The difference of the two proportions *z*-test was used to determine whether the two proportions differed significantly at the 0.05 level of significance.

**Table 1:** Percentage of admissions, grouped by type of primary mental health diagnoses, year of admission (2019, 2020), difference of the two proportions (*z*-score) and *P*

Primary mental health diagnosis	Year			
	2019	2020	*z*-score	*P*
Alcohol disorder	5.0	2.3	1.927	0.054
Anxiety	4.5	2.3	1.610	0.107
Long-term patient	1.6	6.3	3.323	0.001
Depression	10.2	5.7	2.231	0.026
Self-harm/suicidal ideation	11.8	23.2	4.071	<0.001
Mania	2.1	4.3	1.698	0.089
Psychosis	17.6	20.9	1.142	0.254
Substance use disorder	26.2	13.8	4.194	<0.001
Suicidal attempt	7.1	8.3	0.620	0.535
Other	13.9	12.9	0.403	0.689

χ^2^(10) = 54.565, *P* < 0.001.

The primary mental health diagnosis indicating the need for admission to hospital varied between both time periods. The proportion of patients in total presenting with self-harm and suicidal ideation increased from 11.8% in 2019 to 23.2% in 2020. This finding was found to be statistically significant with a *z*-score of 4.071 and a *P*-value of <0.001. Additionally, there was a significant reduction in admissions with substance use disorder, which in 2020 (13.8%) were halved when compared with those in 2019 (26.2%). This was confirmed with a *z*-score of 4.194 and a *P*-value of <0.001. Admissions for alcohol use disorder in 2020 (2.3%) were also halved when compared with those in 2019 (5%), albeit giving a borderline *P*-value of 0.054 and a *z*-score of 1.927. Significant reduction was also identified for primary depressive disorders, which decreased from 10.2% in 2019 to 5.7% in 2020 (*P* = 0.026, *z*-score = 2.231) (Table 1 and Fig. 2).

The differences for other mental health diagnoses were not found to be statistically significant and are as follows: an increase in suicide attempts, from 7.1% in 2019 to 8.3% in 2020 (*P* = 0.535); an increase in admissions of people presenting with psychotic phenomena (17.6% in 2019 to 20.9% in 2020, *P* = 0.254) and manic features (2.1% in 2019 to 4.3% in 2020, *P* = 0.089); and a reduction in anxiety disorders from 4.5% in 2019 to 2.3% in 2020 (*P* = 0.107).

In our data (Table 1 and Fig. 2), the variable ‘other’ refers to presenting complaints that do not have a specific ICD-10 diagnosis and/or occurred in too small numbers to be represented individually, including violent behaviour/aggression; behavioural and psychological symptoms of dementia, intellectual disability, and child and adolescent mental health conditions; methadone and other psychotropic treatment adjustments; social reasons including homelessness; personality disorders; stress-related disorders; bizarre behaviour requiring further observation; forensic admissions and more.

The term ‘long-term patients’ referred to two subgroups of patient admissions. One group consists of individuals who were already resident in the long-term care wings of the mental health facility, usually returning from a medical admission, a surgical procedure or other investigations carried out at our national medical hospital, Mater Dei Hospital. The other group includes those individuals who are community-dwelling, with conditions that require a degree of formal caregiving (for example care homes, hostels) and are often accompanied by psychiatric or behavioural sequelae. Such conditions include intellectual disabilities, severe enduring mental illness and neurocognitive disorders. There were 6 admissions in 2019 and 22 admissions in 2020 from this patient group. Although the total number of patients admitted varied greatly, the distribution of patients according to the abovementioned subgroups was similar (2019: 50% readmissions from long-term care wings, 50% from the community; 2020: 54.5% readmissions from long-term care wings, 45.5% from the community). Similar conditions were observed. In 2019, 16.7% of patients were discharged back into community long-term care, whereas in 2020 10% were discharged from hospital to community long-term care. For further information, kindly refer to the Supplementary Table 4.

The sharp increase in admissions occurring specifically in May 2020 (as shown in Fig. 1) is further elaborated by the figures in the Supplementary material. The most common mental health diagnoses remain self-harm/suicidal ideation (25.3%), followed by psychosis (20.7%), suicide attempts (9.2%) and mania (3.4%).

### Mental Health Act status

In 2020, there was a higher proportion of involuntary admissions using the Mental Health Act (increasing from 27.5% in 2019 to 35.2% in 2020) with an associated lower proportion of voluntary admissions, up to a 7.7% difference between both years. Percentage differences were found to be statistically significant (χ^2^(1) = 4.904, *P* = 0.027).

### Length of stay

During the time period considered in our study, around half of all admissions in both years spent a period of 0–10 days in hospital (50.8% in 2019 and 49.2% in 2020). The finding that is of particular interest is the distribution of the other half of admission stays. Notably, in 2020, there was a higher percentage that spent between 11 and 20 days in hospital (24.9% *v*. 17.3% in 2019) or even longer – between 21 and 30 days (11.8% *v*. 8.4% in 2019). Percentage differences were also found to be statistically significant (χ^2^(6) = 31.306, *P* < 0.001).

Measures of central tendency were used to compare the length of stay for both time periods. The duration distributions in 2019 and 2020 were found to be considerably right-skewed. Therefore, a Mann–Whitney test was applied to compare the differences between the median duration of 2019 (10 days) with that of 2020 (11 days) and this was not found to be significant (*U* = 57 798.5, *P* = 0.860). It was deemed inappropriate to compare mean durations of length of stay because the longest admissions in 2020 could not be captured in our data, in view of them being ongoing, making mean comparisons between the 2 years impossible.

### Logistic regression model

The chi-squared test identified six variables that were significantly related to year of admission. These included the month of admission, age group, gender, primary mental health diagnosis, length of stay and use of the Mental Health Act. A logistic regression model was fitted to relate year of admission to the six aforementioned predictors (Table 2).

**Table 2:** Logistic regression model^a^

Effect	Model fitting criteria, −2 log likelihood of reduced model	Likelihood ratio tests		
		χ^2^	d.f.	*P*
Month of admission	620.671	8.288	3	0.040
Age group	612.902	0.519	2	0.771
Gender	618.037	5.654	1	0.017
Mental Health Act status	619.592	7.210	3	0.066
Primary mental health diagnosis	639.302	26.919	9	0.001
Length of stay	640.401	28.018	6	<0.001

a. Year of admission is the dependant variable and the six variables detailed in the row headings are the predictors.

The logistic regression model identified four significant predictors, which included length of stay (*P* ≤ 0.0001), primary mental health diagnosis (*P* = 0.001), gender (*P* = 0.017) and month of admission (*P* = 0.040). However, Mental Health Act status and age group were not found to be statistically significant (Table 2).

When evaluating odds ratios of mental health hospital admissions in 2020, compared with 2019, females were more likely than males to receive a mental health hospital admission in 2020 and had a higher presentation of self-harm/suicidal ideation.

## Discussion

### Main findings

Although we recognise that other researchers have looked at similar data in their countries, focusing on specific regions,^10^ to our knowledge, this is the first study looking at near-nationwide data of psychiatric hospital admissions during the first wave of the SARS-CoV-2 pandemic. This was possible because of the country's small size (around 500 000) and centralised care through a single in-patient mental health facility.

When considering the absolute number of admissions over this 13-week period, 350 people were admitted in 2020, compared with 401 in 2019. This is in keeping with various international publications reporting an overall reduction in mental health hospital admissions during the initial COVID-19 period.^10–12^

### Interpretation of our findings

Some theories may be posited as to why less people sought in-patient mental healthcare during this period. These include the reluctance to engage with health services because of fear of infection with the virus; a consequent increase in the tolerance threshold of mental health symptoms in both patients and relatives; and a reflection of the marked national investment in community mental health services and telepsychiatry, together with the setting up of a service (the aforementioned admissions and referrals team) with the primary objective of mitigation of people requiring in-patient management. These mitigations included provision of 24 h telephone follow-up for patients seen in an emergency thought to require urgent psychiatric input and ongoing follow-up with referral into secondary care as required; provision of telephone advice to primary healthcare physicians in the management of patients with less complex psychiatric cases; facilitating the transition of more complex patients between primary care and secondary care (i.e. community mental healthcare) and acting as gatekeeping service for any patients thought to require in-patient management.

A point worth noting from our observations is the number of weekly admissions between week 6 and 13 (which coincide with mid-April to May). In this period, we observed a rise in the number of admissions, which in May 2020 even surpassed the admissions in May 2019. Also, the increase in admissions corresponded with a deceleration in the rate of increase of COVID-19 cases. These results substantiate the theory of not engaging through fear of contagion, as when there was a period which was perceived by the public as ‘safer’ because of the reduction in COVID cases, engagement with mental health services increased.

Another hypothesis is that the increase in May 2020 admissions might be attributed to individuals whose mental health had deteriorated to a point where they were unable to postpone receiving in-patient care any longer. This was expected in particular in people with pre-existing mental health conditions, as they are at an increased risk of decompensation and overall worse prognosis in the current strenuous conditions, through difficulties in timely access to mental health services and reduced emotional reserve in coping with stress.^13,14^

When reviewing the age demographics in the samples obtained, one can appreciate that the distribution of the admissions in 2020 remains similar to those in 2019, in that the majority of these are younger than 40 years of age. However, there was also a modest increase in admissions over 60 years of age. Even though this result was not found to be statistically significant, it is a finding worth highlighting, as it was a foreseen outcome of this pandemic, where older people were deemed to be at a disproportionate risk for both physical and mental health complications of COVID-19.^15^ A higher number of psychogeriatric hospital admissions have in fact been reported in other publications.^16^ Social factors that exacerbate the risk of psychological sequelae in this cohort include exacerbation of social isolation through public health directives,^17^ challenges with technological literacy and public transport restrictions.^18^ One must also mention people with dementia and the marked difficulties they have faced in understanding the concept of a pandemic and the need to abide by infection control measures, leading to further physical and mental health risks and caregiver burnout.^19^

### Socioeconomic contributors

It has been widely published that the COVID-19 pandemic has had a detrimental effect on the general population. Psychological manifestations directly related to the pandemic have included fear, avoidance, obsessions with hygiene, fear of death (thanatophobia), fear of isolation and stigmatisation.^20^ However, there have been a multitude of socioeconomic contributors that have further exacerbated this deterioration and risk to self. Some examples include: economic strain through business closures and restriction of practices in various industries, with fear of recession and increasing rates of unemployment and financial hardship; worsened social isolation through public health measures of social distancing and discontinuation of informal support networks such as community and/or religious activities; and difficulties in accessing mental health services as described earlier.^21^

### Changing patterns in mental health presentations

From the limited research that exists with regards to epidemics/pandemics and suicide (for example the 1918 influenza, severe acute respiratory syndrome, Middle East respiratory syndrome, Zika, Ebola and influenza A virus subtype H1N1), there appeared to be a strong association between such events and a rise in suicidal behaviour, thought to be in part because of exacerbation of pre-existing mental illness related to the fears about the outbreak.^22^ It is based on research from past pandemics that some have predicted that suicide rates would increase during the COVID-19 pandemic.^23^ In light of the above, the results we derived from our data on primary mental health diagnoses further support the postulations outlined previously. One of the most important findings was the significant surge in hospital admissions primarily because of self-harm and suicidal ideation, which were the only mental health diagnoses found to be significantly higher following *z*-test analysis (see Table 1 and Fig. 2). This phenomenon was especially notable in May 2020, where severe mental health presentations were most prominent. These were also accompanied by a reduction in admissions because of depression and/or anxiety (without active risk to self) and a marked decrease in substance (and alcohol) use disorder presentations.

The change in mental health presentations may partially explain the statistically significant increase in female admissions between 2019 and 2020. This is because literature has shown that whereas men are more likely to present with psychosis or aggression and have diagnoses of schizophrenia and substance use disorder, women are more likely to present with self-harming behaviours and have a background of mood disorders with comorbid psychiatric conditions such as personality disorders.^24^

The rise in use of the Mental Health Act also reflects the occurrence of serious psychiatric presentations that may affect insight into the condition and thus requiring involuntary admission, as well as the overall increase in lengths of stay in 2020 when compared with 2019. However, a factor that could have contributed to the prolongation of stays is the restructuring of certain community and social services in the light of the COVID pandemic, which led to unforeseen delays in discharge planning from hospital.

One can appreciate these results as possibly reflecting a rise in ‘inevitable’ admissions because of severity of illness or risk posed to self and/or others requiring more intensive in-patient treatment and management, complemented by a drop in admissions that were deemed ‘unnecessary’, or rather, amenable to less intensive management in the community.

Nevertheless, this also questioned the ultimate end-effect of the restructured community mental health services. Although the perceived augmentation of community mental health intervention, as well as the bolstering of addiction services, offered by the National Health Service (such as a detox clinic) and non-governmental organisations^25^ may have led to better management of primary anxious and depressive disorders and substance use disorder, these adaptations may not have been sufficient or adequate to support those individuals who were in crisis.

Another possible shortcoming of the restructured community services was perceptible in the increase of long-term patient admissions to hospital. The scaling back or suspension of community services such as assertive outreach (for severe and enduring mental illness) or other specialist services that used to provide domiciliary visits (for intellectual disabilities and neurocognitive disorders) led to reduced support of these patient populations with consequent deterioration and need for in-patient stabilisation. An increased number of this patient group also likely remained in hospital because of the logistic challenges faced in discharging patients back into community long-term care facilities because of the pandemic.

These insights offer potential avenues for further research into the out-patient component of mental health services that may lead to amelioration in the aforementioned "challenges".

### Limitations

A limitation that was encountered in this study was during the data-collection process. This was because electronic patient data records have still not been implemented in Malta. Therefore, it was difficult to obtain additional data from earlier years for detailed comparisons with previous admission rates in such a short period of time. Furthermore, some admissions had to be omitted from data analysis as the data available were incomplete.

The primary mental health diagnosis was identified according to what was recorded on discharge summaries. In some cases, albeit this was a minority, the clinical diagnosis listed did not always follow the ICD-10 classification criteria as rigorously as required, thus reducing the quality of the data available. This situation was rectified by reviewing those patients’ clinical files and eliciting a diagnosis conforming to the ICD-10 criteria from the symptomatology described in their presentation.

We used hospital admissions as a measure to assess the effects COVID-19 had on the nation's mental health hospital admissions. However, we understand that these results do not capture the impact on mental health as a whole. The full implications are likely to be a lot more complex and widespread in the community. Analysing out-patient data will have likely shed some more light and given an overall better understanding; however, this was not available to us and therefore we can only hypothesise. Furthermore, there might have been psychological sequelae that were not identified because they might not have come to the attention of mental health professionals.

One of the recommended statistical procedures to assess the effects of COVID-19 on mental health hospital admissions is to use interrupted time series. However, this methodology requires long-term observations before and after the interruption. In this study, monthly admissions were recorded from 7 March to 4 June, yielding two data points before the interruption (readings in May and April of 2019) and two data points after the interruption (readings in May and April of 2020). Monthly admissions in March and June would have had to be excluded because admission frequencies do not cover whole months. Owing to the sparse information before and after the interruption, interrupted time series analysis could not be carried out.

### Implications

Our population-based study has given a strong indication that the occurrence of the COVID-19 pandemic and the eventual imposition of restrictions by health authorities had an effect on the nation's mental health; our findings highlight an increase in admissions to hospital for people with serious mental illnesses such as harm to self and suicidal behaviour and a reduction in admissions for people with primary anxiety or depression disorders and substance use disorders. Our results confirm the urgency to safeguard mental health with the same attention and vigour as physical health has been. One year on, several countries are still presently battling the spread of COVID-19 and its emergent variants as vaccines are being rolled out to the public, while issuing further restrictions and lockdowns and revising policies. In addition to this, mental health services have undergone marked changes and adaptations in order to face the pressures that the COVID-19 pandemic has posed. As these pressures are predicted to continue in the coming months (and years), there is a pressing need to further strengthen psychiatric services, so as to be able to effectively provide for and nurture the needs of the population.

This study starts to pave the way for further local research into subsequent waves of the pandemic; through comparison of data it will be possible to identify whether the national mental health service continues to adapt to COVID-19 by learning from these findings.

## Data Availability

The authors confirm that the data supporting the findings of this study are available within the article and/or its Supplementary material.

## Supplementary material

For supplementary material accompanying this paper visit http://dx.doi.org/10.1192/bjo.2021.975. click here to view supplementary material
