# Supplementary material for: Effect of COVID-19 pandemic on mental health hospital admissions: comparative population-based study
Source: BJPsych Open. 2021 Aug 3;7(5):e141. doi: 10.1192/bjo.2021.975 (PMC8365103; doi:10.1192/bjo.2021.975)
Supplement: Supplementary file 1 [file S2056472421009753sup001.docx]

**Annexes (Supplementary material)**

|  | | | Year | |
| --- | --- | --- | --- | --- |
|  |  |  | 2019 | 2020 |
| Date of admission | March | Count | 108 | 58 |
|  |  | Percentage | **26.9%** | **16.6%** |
|  | April | Count | 134 | 98 |
|  |  | Percentage | **33.4%** | **28.0%** |
|  | May | Count | 135 | 174 |
|  |  | Percentage | **33.7%** | **49.7%** |
|  | June | Count | 24 | 20 |
|  |  | Percentage | **6.0%** | **5.7%** |
| Total | | Count | 401 | 350 |
|  |  | Percentage | **100.0%** | **100.0%** |

X^2^(3) = 22.573, p < 0.001

**Table 1: Comparison rates of psychiatric admissions to Mount Carmel Hospital between March and June 2019 with the same time period in 2020.**


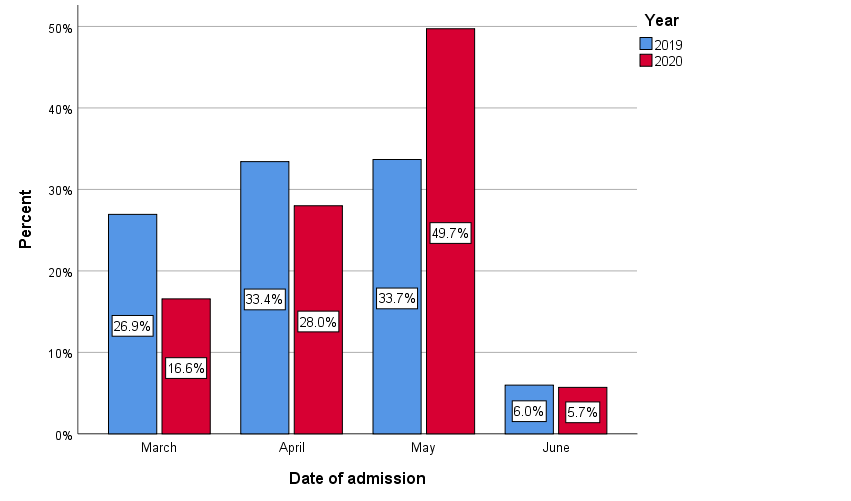


**Figure 1: Total number of admissions to Mount Carmel Hospital occurring between March and June 2019 compared to the same time period in 2020. *(Date of Admission – x-axis vs. Percent Admissions – y-axis).***

|  | | | Year | | Total |
| --- | --- | --- | --- | --- | --- |
|  |  |  | 2019 | 2020 |  |
| Age | 40 years or less | Count | 224 | 176 | 400 |
|  |  | Percentage | **57.0%** | **50.3%** | **53.8%** |
|  | 41-60 years | Count | 124 | 115 | 239 |
|  |  | Percentage | **31.6%** | **32.9%** | **32.2%** |
|  | More than 60 years | Count | 45 | 59 | 104 |
|  |  | Percentage | **11.5%** | **16.9%** | **14.0%** |
| Total | | Count | 393 | 350 | 743 |
|  |  | Percentage | **100.0%** | **100.0%** | **100.0%** |

X^2^(2) = 5.513, p = 0.063

**Table 2: Comparison rates of age groups in admissions occurring between March and June 2019 with the same time period in 2020.**

|  | | | Year | |
| --- | --- | --- | --- | --- |
|  |  |  | 2019 | 2020 |
| Gender | Female | Count | 116 | 140 |
|  |  | Percentage | **28.9%** | **40.0%** |
|  | Male | Count | 285 | 210 |
|  |  | Percentage | **71.1%** | **60.0%** |
| Total | | Count | 401 | 350 |
|  |  | Percentage | **100.0%** | **100.0%** |

X^2^(1) = 10.197, p < 0.001

**Table 3: Comparison rates of gender differences in admissions occurring between March and June 2019 with the same time period in 2020.**


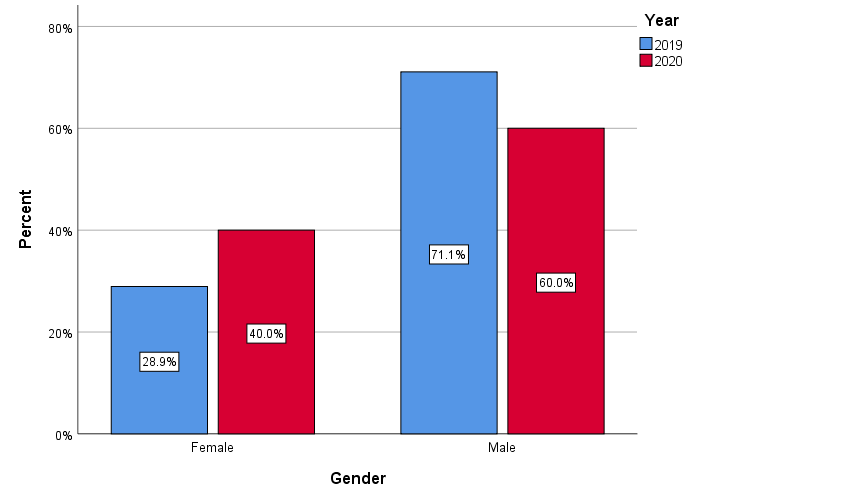


**Figure 2**: **Comparison rates of gender differences in admissions occurring between March and June 2019 with the same time period in 2020.** *Gender (x-axis) vs. Percentage admissions (y-axis).*

|  |  | **Year** | |
| --- | --- | --- | --- |
|  |  | **2019** | **2020** |
| **Total number of patients** | | **6** | **22** |
| Age | 0-40 | 1 | 1 |
|  | 41-60 | 1 | 4 |
|  | >60 | 4 | 17 |
| Gender | Male | 4 | 12 |
|  | Female | 2 | 10 |
| Original Place of Stay | Mount Carmel Hospital | 3 | 12 |
|  | Community Home | 2 | 6 |
|  | Private residence | 1 | 4 |
| Psychiatric diagnosis | Bipolar Disorder | 1 | 2 |
|  | Dementia | 1 | 2 |
|  | Depression | - | 3 |
|  | Huntington's Disease | - | 4 |
|  | Learning Disability | 2 | 4 |
|  | Schizophrenia | 2 | 7 |
| Discharge location | Mount Carmel Hospital | 5 | 20 |
|  | Long-term care facility | 1 | 2 |

**Table 4: Comparison details of long-term patients admitted to Mount Carmel Hospital between 2019 and 2020, containing demographic details, original place of stay, primary psychiatric diagnosis and discharge location by the end of the study period.**

|  | | | Year | |
| --- | --- | --- | --- | --- |
|  |  |  | May 2019 | May 2020 |
| Primary Mental Health Diagnosis | Alcohol Use Disorder | Count | 8 | 6 |
|  |  | Percentage | **6.2%** | **3.4%** |
|  | Anxiety | Count | 6 | 6 |
|  |  | Percentage | **4.7%** | **3.4%** |
|  | Long-term patient | Count | 2 | 11 |
|  |  | Percentage | **1.6%** | **6.3%** |
|  | Depression | Count | 15 | 7 |
|  |  | Percentage | **11.6%** | **4.0%** |
|  | DSH/Suicidal Ideation | Count | 23 | 44 |
|  |  | Percentage | **17.8%** | **25.3%** |
|  | Mania | Count | 3 | 6 |
|  |  | Percentage | **2.3%** | **3.4%** |
|  | Psychosis | Count | 20 | 36 |
|  |  | Percentage | **15.5%** | **20.7%** |
|  | Substance Use Disorder | Count | 26 | 25 |
|  |  | Percentage | **20.2%** | **14.4%** |
|  | Suicide attempt | Count | 9 | 16 |
|  |  | Percentage | **7.0%** | **9.2%** |
|  | Other | Count | 17 | 17 |
|  |  | Percentage | **13.2%** | **9.8%** |
| Total | | Count | 129 | 174 |
|  |  | Percentage | **100.0%** | **100.0%** |

X^2^(9) = 17.256, p = 0.045

**Table 5: Comparison rates of the primary mental health diagnosis that necessitated an admission specifically between May 2019 and May 2020.** *DSH = Deliberate Self-harm.*

|  | | | Year | | Total |
| --- | --- | --- | --- | --- | --- |
|  |  |  | 2019 | 2020 |  |
| MHA Status | MHA sectioned | Count | 102 | 117 | 219 |
|  |  | Percentage | **27.5%** | **35.2%** | **31.2%** |
|  | Voluntary | Count | 269 | 215 | 484 |
|  |  | Percentage | **72.5%** | **64.8%** | **68.8%** |
| Total | | Count | 371 | 332 | 703 |
|  |  | Percentage | **100.0%** | **100.0%** | **100.0%** |

X^2^(1) = 4.904, p = 0.027

**Table 6: Comparison rates of the Mental Health Act status of admissions occurring between March and June 2019 with the same time period in 2020.** *MHA = Mental Health Act*


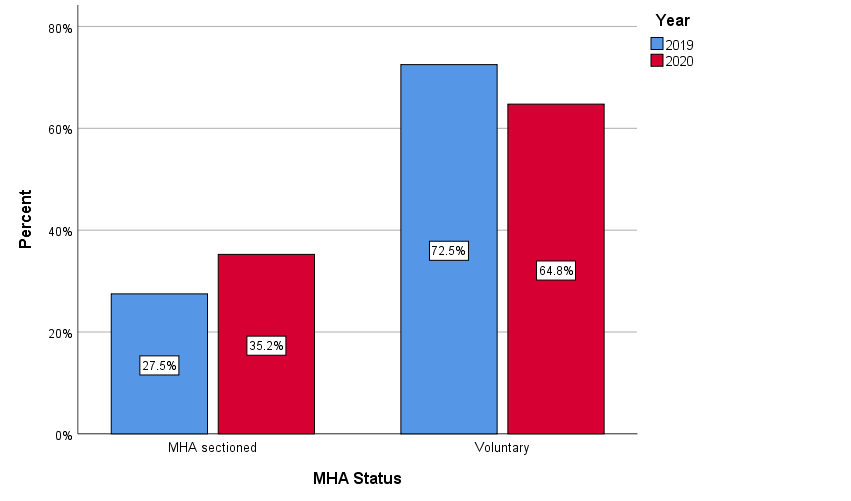


**Figure 3**: **Comparison rates of the Mental Health Act status of admissions occurring between March and June 2019 with the same time period in 2020. *MHA Status (x-axis) vs. Percentage admissions (y-axis).*** *MHA = Mental Health Act.*

|  | | | Year | |
| --- | --- | --- | --- | --- |
|  |  |  | 2019 | 2020 |
| Length of stay (days) | 0-10 | Count | 194 | 150 |
|  |  | Percentage | **50.8%** | **49.2%** |
|  | 11-20 | Count | 66 | 76 |
|  |  | Percentage | **17.3%** | **24.9%** |
|  | 21-30 | Count | 32 | 36 |
|  |  | Percentage | **8.4%** | **11.8%** |
|  | 31-40 | Count | 21 | 18 |
|  |  | Percentage | **5.5%** | **5.9%** |
|  | 41-50 | Count | 17 | 16 |
|  |  | Percentage | **4.5%** | **5.2%** |
|  | 51-100 | Count | 33 | 9 |
|  |  | Percentage | **8.6%** | **3.0%** |
|  | More than 100 | Count | 19 | 0 |
|  |  | Percentage | **5.0%** | **0.0%** |
| Total | | Count | 382 | 305 |
|  |  | Percentage | **100.0%** | **100.0%** |

X^2^(6) = 31.306, p < 0.001

**Table 7: Comparison rates of the total length of stay for admissions occurring between March and June 2019 with the same time period in 2020.**


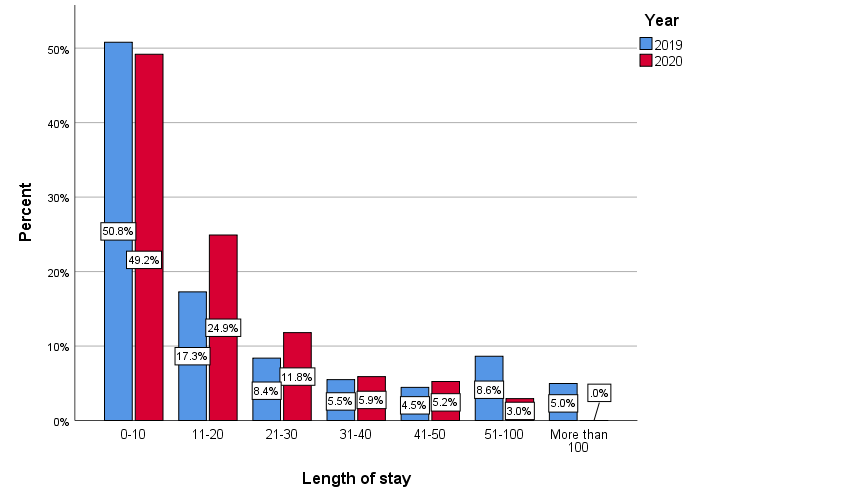


**Figure 4**: **Comparison rates of the total length of stay for admissions occurring between March and June 2019 with the same time period in 2020. *Length of stay (x-axis) vs. percentage admissions (y-axis).***

|  | Year | | Statistic |
| --- | --- | --- | --- |
| Length of stay | 2019 | Median | 10.00 |
|  |  | Range | 471.00 |
|  |  | Interquartile Range | 25.25 |
|  | 2020 | Median | 11.00 |
|  |  | Range | 96.00 |
|  |  | Interquartile Range | 16.00 |

**Table 8: Comparison of the measures of central tendency for 2019 and 2020 hospitalisation duration.**


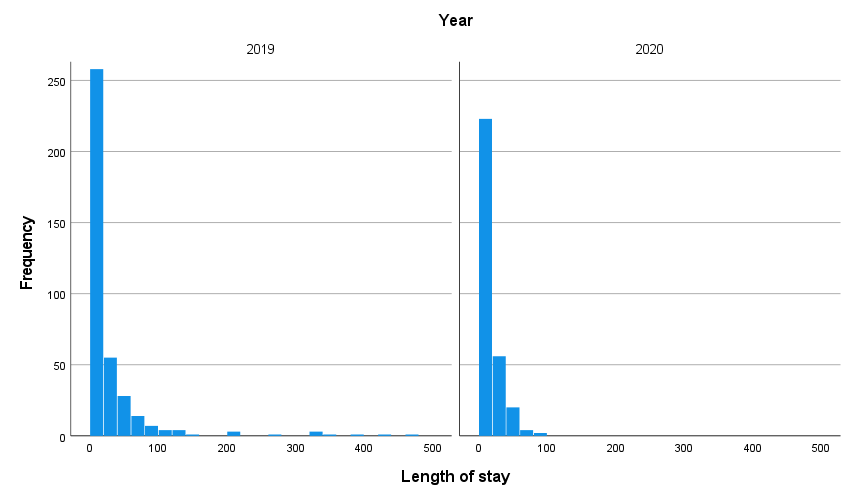


**Figure 5: Frequency graph of length of stay of hospitalisation (x-axis) vs. frequency admissions in both 2019 and 2020, showing marked right-skewness in distribution.**

| **Mann Whitney test** | | | | | |
| --- | --- | --- | --- | --- | --- |
|  | Year | Sample size | Median Duration | Range | Interquartile Range |
| Length of stay | 2019 | 382 | **10** | 471 | 25.25 |
|  | 2020 | 305 | **11** | 96 | 16.00 |

U = 57798.5, p = 0.860

**Table 9: Mann Whitney test to measure the difference between the two median hospital durations in 2019 and 2020.**

| **Odds ratios** | | | | | | | |
| --- | --- | --- | --- | --- | --- | --- | --- |
| Year of analysis - 2020 | | B | Std. Error | Wald | df | P-value | Odds ratio |
|  | Month of admission = March | -0.285 | 0.428 | 0.443 | 1 | 0.506 | 0.752 |
|  | Month of admission = April | -0.238 | 0.414 | 0.329 | 1 | 0.566 | 0.788 |
|  | Month of admission = May | 0.255 | 0.408 | 0.389 | 1 | 0.533 | 1.290 |
|  | Month of admission = June | 0 | . | . | 0 | . | . |
|  | Gender = Female | 0.399 | 0.177 | 5.091 | 1 | **0.024** | **1.491** |
|  | Gender = Male | 0 | . | . | 0 | . | . |
|  | Primary MH Dx = Alcohol dependence | -0.711 | 0.492 | 2.091 | 1 | 0.148 | 0.491 |
|  | Primary MH Dx = Anxiety | -0.589 | 0.451 | 1.702 | 1 | 0.192 | 0.555 |
|  | Primary MH Dx = Long-term patient | 0.023 | 0.948 | 0.001 | 1 | 0.981 | 1.023 |
|  | Primary MH Dx = Depression | -0.752 | 0.396 | 3.601 | 1 | 0.058 | 0.471 |
|  | Primary MH Dx = DSH / Suicidal ideation | 0.584 | 0.293 | 3.965 | 1 | **0.046** | **1.793** |
|  | Primary MH Dx = Mania | 0.359 | 0.520 | 0.475 | 1 | 0.491 | 1.432 |
|  | Primary MH Dx = Psychosis | 0.130 | 0.288 | 0.204 | 1 | 0.652 | 1.139 |
|  | Primary MH Dx = Substance misuse | -0.545 | 0.288 | 3.599 | 1 | 0.058 | 0.580 |
|  | Primary MH Dx = Suicidal attempt | 0.049 | 0.359 | 0.019 | 1 | 0.891 | 1.050 |
|  | Primary MH Dx = Other | 0 | . | . | 0 | . | . |
|  | Length of stay = More than 100 days | -1.660 | 0.525 | 9.998 | 1 | **0.000** | **0.190** |
|  | Length of stay = 51-100 days | -0.987 | 0.404 | 5.953 | 1 | **0.015** | **0.373** |
|  | Length of stay = 41-50 days | 0.053 | 0.382 | 0.019 | 1 | 0.890 | 1.054 |
|  | Length of stay = 31-40 days | 0.040 | 0.356 | 0.013 | 1 | 0.910 | 1.041 |
|  | Length of stay = 21-30 days | 0.233 | 0.280 | 0.693 | 1 | 0.405 | 1.263 |
|  | Length of stay = 11-20 days | 0.287 | 0.212 | 1.834 | 1 | 0.176 | 1.332 |
|  | Length of stay = 0-10 days | 0 | . | . | 0 | . | . |
| The reference category is: 2019 | | | | | | | |

**Table 10: Odds ratio of mental health hospitalisations comparing 2019 with 2020 using the four significant predictors.**

Odds ratios that are significantly larger than 1 indicate a significant increase in prevalence in 2020 compared to 2019. So the percentage increments in the number of **female patients** and the number of **DSH / Suicidal ideation** presentations are significant.

On the other hand, odds ratios that are significantly smaller than 1 indicate a significant decrease in prevalence in 2020 compared to 2019. Therefore, admissions with a length of stay of **more than 50 days** were significantly less prevalent in 2020 compared to 2019, as some admissions during COVID year were still ongoing during the data collection of our study.
